# Reply to “Comparisons of Within-Group Instead of Between-Group Affect the Conclusions. Comment on: Changes in Weight and Substrate Oxidation in Overweight Adults Following Isomaltulose Intake during a 12-Week Weight Loss Intervention: A Randomized, Double-Blind, Controlled Trial. *Nutrients* 2019, *11*(10), 2367”

**DOI:** 10.3390/nu12082339

**Published:** 2020-08-05

**Authors:** Helen Lightowler, Lisa Schweitzer, Stephan Theis, Christiani Jeyakumar Henry

**Affiliations:** 1 Oxford Brookes Centre for Nutrition and Health, Department of Sport, Health Sciences and Social Work, Faculty of Health and Life Sciences, Oxford Brookes University, Headington Campus, Gipsy Lane, Oxford OX3 0BP, UK; p0073016@brookes.ac.uk; 2 BENEO-Institute, BENEO GmbH, Wormser Straße 11, 67283 Obrigheim/Pfalz, Germany; lisa.schweitzer@beneo.com (L.S.); stephan.theis@beneo.com (S.T.); 3 Clinical Nutrition Research Centre (CNRC), Singapore Institute for Clinical Sciences (SICS), Agency for Science, Technology and Research (A*STAR) and National University Health System, Centre for Translational Medicine, 14 Medical Drive #07-02, MD 6 Building, Yong Loo Lin School of Medicine, Singapore 117599, Singapore

**Keywords:** isomaltulose, glycemic index, overweight, weight management

We thank the authors Vorland, Kyle and Brown for their interest in our paper and their comments. We are very pleased to respond to their inquiry and highly appreciate the compliments paid on the conduct of the study [1].

Our study was a 3-month randomized controlled weight-loss intervention in overweight and obese participants [2]. The primary outcome was body weight loss following an energy-reduced diet containing foods sweetened with Palatinose™ or sucrose. We aimed to determine whether a diet containing foods sweetened with Palatinose™ promotes weight loss as such, in addition to differences in body weight loss between the groups.

We also conducted a robust statistical analysis which was comprehensibly described in the Materials and Methods section. We found a significant body weight loss after 3 months when compared to baseline which was independent of the group. However, as reported in the publication, we did not observe a statistically significant between-group difference regarding the primary outcome i.e., body weight change, which was −3.2 ± 2.9 kg in the Palatinose™ group and −2.1 ± 2.6 kg in the sucrose group over the 3-month period. As correctly pointed out by Vorland et al., significance level for the primary outcome was set at *p* < 0.05. The effect size, variability as well as all respective *p*-values were clearly and transparently described throughout the manuscript, which is in line with the American Statistical Association (ASA) statement of ‘*full reporting and transparency*’ [3]. Hence, we respectfully disagree with Vorland et al. that we reported results distortedly as we did not hide any information from the reader. We agree that ‘*Accurate presentation of the results of a randomized controlled trial (RCT) is the cornerstone of the dissemination of the results…*’ as described in the cited reference by Boutron et al. [4]. We would like to emphasize that we neither intend to obscure any results nor mislead the readers through this publication. Rather we strived to give a complete and transparent view on the results of this trial. In our view, this includes the acknowledgement of all results.

Interpretation of trial results is not a simple, straightforward process. On occasions, some disagreement may inevitably arise. Even among authors, no objective measure exists for the subjective component of interpretation. In this respect, the ASA stated that ‘*Scientific conclusions and business or policy decisions should not be based only on whether a p-value passes a specific threshold.*’ [3], and advised taking the entire context into account when interpreting results. Consequently, we discussed the findings intensively with the literature and drew conclusions considering several contextual factors, e.g., relevant external evidence, validity of assumptions, study design / diet, clinical relevance.

A difference of 1 kg body weight reduction is suggested to be clinically meaningful. According to the AHA/ACC/TOS (American Heart Association / American College of Cardiology / The Obesity Society) Guideline for the Management of Overweight and Obesity, a high strength of evidence for a dose-dependent relationship between the amount of weight loss and the improvement of cardio-metabolic outcomes is reported (e.g., blood lipids, blood pressure, HbA1c etc.) [5].

To express the uncertainty of findings obtained in our study we have used rather cautious phrasing (e.g., ‘*suggest*’ and ‘*may help*’) when interpreting the results. From the letter of Vorland et al. we understand that in their opinion this has, however, not been implemented sufficiently. In order to address the request and to avoid any misunderstanding which may arise for some readers of our publication, we would like to point out that the statement in the abstract could have been even better phrased as ‘*During the 12 weeks, both groups significantly lost weight (p < 0.001), which was −3.2 ± 2.9 kg in the ISO and −2.1 ± 2.6 kg in the SUC group (p = 0.258).*’. Moreover, we could have chosen the wording even better in the discussion, i.e., ‘*…consumption of ISO compared to that of SUC may be more effective at promoting weight loss*’.

In addition, to emphasize the lack of significant between-group effects in the overall conclusion, a clearer and more comprehensive wording would be: ‘*The present weight loss intervention in overweight and obese individuals might suggest that participants on a LGI diet with ISO achieve higher weight loss as well as fat mass loss compared to their counterparts in the SUC group. Yet, for both parameters the between-group difference did not reach the pre-set level of statistical significance.*’.

We value the opportunity to respond to the letter and hope that with the additional explanation we could adequately address the readers´ concern and clarify the interpretation of results.
